# Approach to high quality GaN lateral nanowires and planar cavities fabricated by focused ion beam and metal-organic vapor phase epitaxy

**DOI:** 10.1038/s41598-018-25647-7

**Published:** 2018-05-08

**Authors:** Galia Pozina, Azat R. Gubaydullin, Maxim I. Mitrofanov, Mikhail A. Kaliteevski, Iaroslav V. Levitskii, Gleb V. Voznyuk, Evgeniy E. Tatarinov, Vadim P. Evtikhiev, Sergey N. Rodin, Vasily N. Kaliteevskiy, Leonid S. Chechurin

**Affiliations:** 1 Department of Physics, Chemistry and Biology (IFM), Linköping University, S-581 83 Linköping, Sweden; 2 St-Petersburg Academic University Khlopina 8/3, 194021 St. Petersburg, Russian Federation; 3 ITMO University, Kronverkskiy pr. 49, 197101 St. Petersburg, Russian Federation; 4 Ioffe Institute, Politekhnicheskaya 26, 194021 St. Petersburg, Russian Federation; 5 SHM R&E Center RAS, 194021 St. Petersburg, Russian Federation; 6 Lappeenranta University of Technology, Lappeenranta, FI-53851 Finland

## Abstract

We have developed a method to fabricate GaN planar nanowires and cavities by combination of Focused Ion Beam (FIB) patterning of the substrate followed by Metal Organic Vapor Phase Epitaxy (MOVPE). The method includes depositing a silicon nitride mask on a sapphire substrate, etching of the trenches in the mask by FIB with a diameter of 40 nm with subsequent MOVPE growth of GaN within trenches. It was observed that the growth rate of GaN is substantially increased due to enhanced bulk diffusion of the growth precursor therefore the model for analysis of the growth rate was developed. The GaN strips fabricated by this method demonstrate effective luminescence properties. The structures demonstrate enhancement of spontaneous emission via formation of Fabry-Perot modes.

## Introduction

Nanowires (NWs) based on GaN and related III-N alloys^1,2^ have a huge potential for innovative semiconductor devices such as field effect transistors^3,4^, lasers^5,6^, light emitting diodes^7^, sources of single photons and entangled photon pairs^8,9^ and qubits^10,11^. Development of the technology of nanowire fabrication pave the way for the substitution of so–called “top-down” approach (implying the growth of planar structures followed by etching of unwanted parts of the structures) with “bottom-up” approach when the functional elements are grown in its final form^12,13^. In recent years many advantages have been made in the area of fabrication of nanowires in the form of nano-pillars using vapor-liquid-solid (VLS)^14–16^ and vapor-solid-solid^17,18^ growth mechanisms. Molecular beam epitaxy^19–21^, gas phase epitaxy^22–24^ and magnetron sputter epitaxy^25–28^ are the most often used techniques of nanopillars growth. Such technology allows fabrication of high quality NW exhibiting excellent electric and optical properties, even in the case of substantial mismatch of the layer parameter between NW and substrate material and allows fabrication of III-V NWs on Si substrates^29,30^. Despite recent significant advances in improving growth of semiconductor nanowires it is difficult to control regularity, orientation and arrangement of nanowires for optoelectronic devices. However, for a reliable industrial realization of electronic and optoelectronic devices the demand is not only a formation of high-quality GaN nanowires, but also the possibility of precisely controlling the nanowires geometry and position on the substrate.

In this paper we describe a “bottom-up” approach of fabrication of lateral GaN NWs on a sapphire substrate based on Focused Ion Beam (FIB)^31^ patterning of the substrate followed by Metal-Organic Vapor Phase Epitaxy (MOVPE) technique, which is well developed for growth of III-nitrides^32,33^. Although the approach is very attractive, there is a significant complicity in the production of high quality NWs associated with an enhanced growth rate in selective area MOVPE. To obtain a high crystal quality lateral GaN NWs, the mechanism behind the increased growth rate has to be understood. Thus, here we consider a theoretical model allowing optimized growth regimes, especially the Ga precursor concentration. Accordingly, we present in this work results of our studies of high crystal quality lateral GaN NWs produced by optimized MOVPE process.

## Results and Discussion

**Figure 1:**
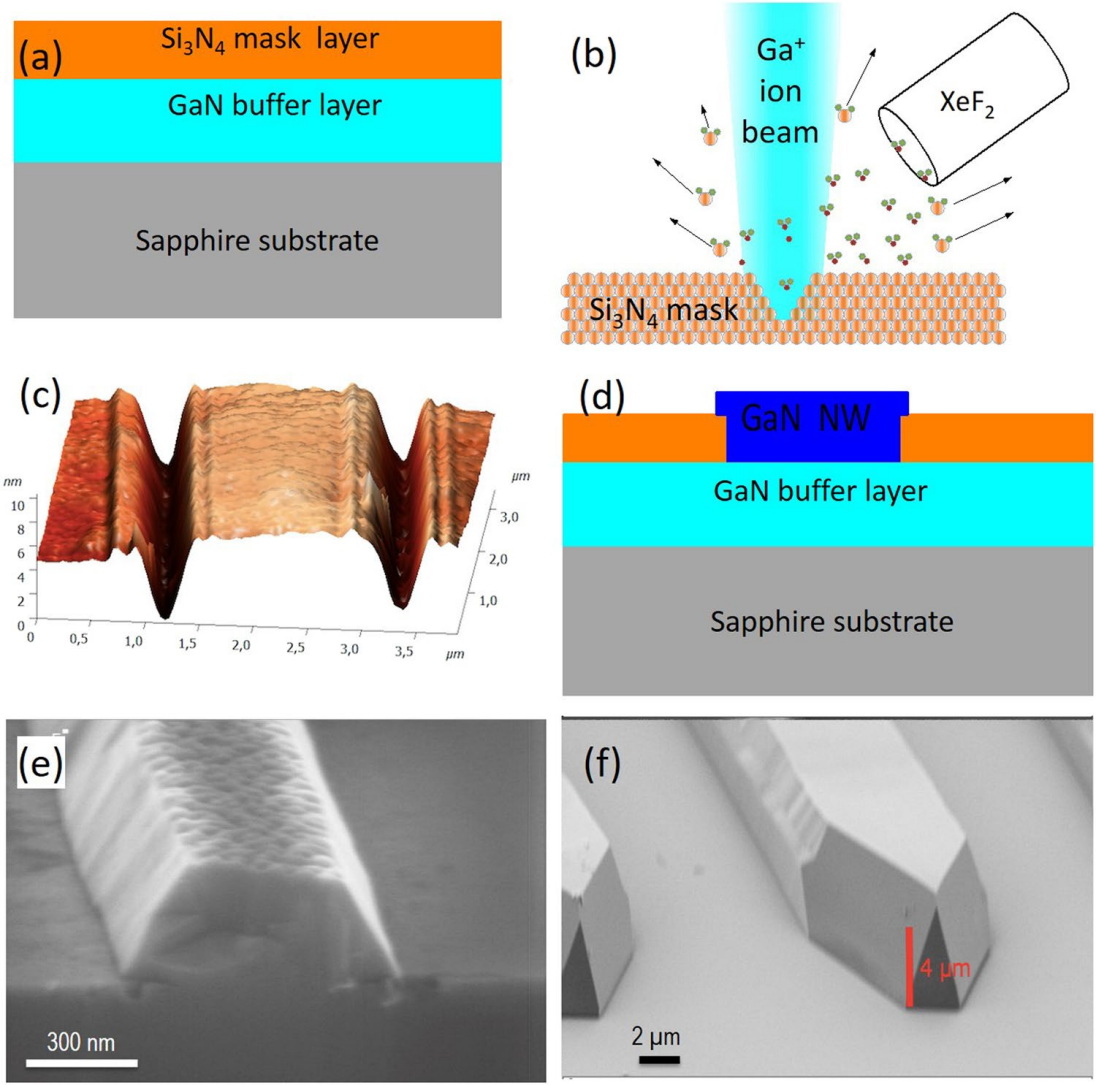
Illustration of fabrication process. (**a**) Sapphire wafer covered by 3 μm GaN doped buffer layer and 5 nm Si_3_N_4_ mask layer; (**b**) FIB etching of mask layer; (**c**) AFM image of 200 nm wide trenches in Si_3_N_4_ mask layer; (**d**) GaN NW grown in the trench by MOVPE. (**e**,**f**) SEM images of the NWs grown on the trenches of different width.

Fabrication process steps to produce lateral GaN NWs are schematically shown in Fig. 1. The MOVPE GaN buffer layer was grown on sapphire substrate and then it was covered by Si_3_N_4_ mask (Fig. 1a). The 200 µm long single trenches and periodic arrays of trenches with the thickness in the interval from 100 to 500 nm were etched by FIB, see Fig. 1b. Figure 1c shows an example of the Atomic Force Microscopy (AFM) image of periodic trenches in the mask. Selective area MOVPE growth was employed on the patterned wafers to fabricate lateral GaN NWs. For more details, see section Methods. Figures 1e,f show an example of NWs grown on trenches of different width. It can be seen that the cross-section of NWs have trapezoid-like shape and the lateral size exceeds the size of the trench due to anisotropic growth of GaN.

It is known that in the selective area MOVPE process, the growth rate increases compared to a conventional planar case due to distortion of the spatial profile of precursor concentration leading to enhanced bulk diffusion complemented by the surface diffusion of precursors from the mask to unmasked area^34^. A boundary layer (where the layer diffusion mass transfer dominates over convention mass transfer)^35^ is formed near the surface of the wafer and the thickness of the boundary layer can be estimated as:

1 $$b=5\ast \sqrt{\frac{\nu g}{U}},$$

where *U* is the gas velocity, *g* - is the horizontal size of the substrate and *v* is the kinematic viscosity. Typical thickness of the boundary layer was about 5 nm for the growth parameters used in our MOCVD process.

An enhancement of the growth rate can be estimated by solving a system of diffusion equations for bulk and for surface transport. Since the length of the NW (200 μm) is much larger than its width (fraction of μm), we can model bulk and surface mass transfer by solving a system of diffusion equations in the plane (XOZ) perpendicular to the axis of NW (Y-axis).

The bulk concentration *C* of the precursor limiting the growth (i.e. TMG) within the boundary layer satisfies the Laplace equation:

2 $$\frac{{\partial }^{2}C}{\partial {x}^{2}}+\frac{{\partial }^{2}C}{\partial {z}^{2}}=0$$

Concentration *C* on the edge of boundary layer corresponds to the concentration in the incoming gas flow *C*_0_:

3 $$C{|}_{z=b}={C}_{0}$$

while for the unmasked area of the substrate, where the precursor quickly absorbs to the surface, the concentration corresponds to zero:

4 $$C{|}_{z=0}=0.$$

**Figure 2:**
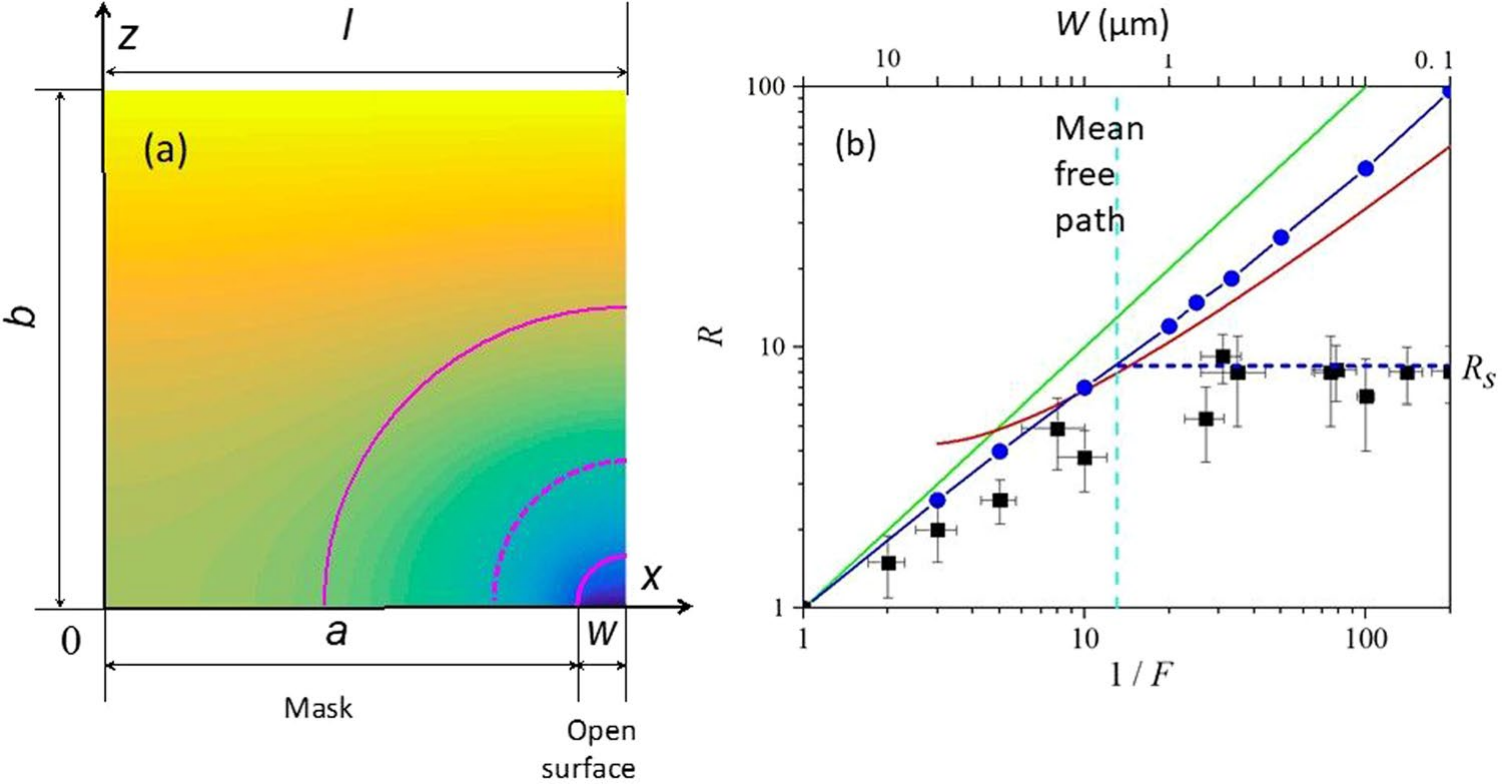
Modelling of the diffusion of growth precursors in the boundary layer under the masked surface. The half of the period of the structure, widths of the mask, open area and thickness of the boundary layer are denoted as *l, a, w* and *b*, respectively. (**a**) Concentration of the growth precursor *C* in the boundary layer over the masked surface calculated using differential equation (Eq. 2) and boundary conditions Eq. (3–8). Solid circular lines shows an isoline of bulk concentration used for development of a simplified analytical model. The dashed circular line indicates the distance corresponding to the mean free path of the precursors in gas (all shown not to scale). (**b**) Dependence of the relative growth rate *R* on the filling factor *F* calculated by Eq. (9) (blue circles), by analytical estimate Eq. (11) (red line) and dependence 1/*F* (green line). The squares show the experimentally obtained values of *R*. Upper top scale shows the corresponding width of the open area *w*. A horizontal dashed line shows the saturation value of the relative growth rate *R*_*S*_, while a vertical dashed line shows the size of the open area corresponding to the mean free path of the precursor in the gas.

Assuming that the growth precursor can be absorbed to and desorbed from the surface of the mask we can introduce a surface concentration of the precursor *C*^*surf*^ = *kC*, where *k* is a coefficient with the dimensionality of length. For the masked area we can write a surface diffusion equation complemented by the precursor flow to or from surface:

5 $$D\frac{\partial C}{\partial z}{|}_{z=0,0 < x < a}={D}^{surf}k\frac{{\partial }^{2}C}{{\partial }^{2}z}{|}_{z=0,0 < x < a}$$

where *D* and *D*^*surf*^ are the bulk and the surface diffusion coefficients, respectively. For the completeness of the system, one also should set the boundary conditions on the left and right boundaries of the modeling area. For the periodic mask with the period 2 *l* due to symmetry reasons, the dependence *C*(*x*) is a symmetric function of *x* when *x* = 0 or *x* = *l* and the boundary condition is:

6 $$\frac{\partial C}{\partial x}{|}_{x=0}=\frac{\partial C}{\partial x}{|}_{x=1}=0$$

where the points coordinates *x* = 0 and *x* = 0 correspond to centers of unmasked and masked areas, respectively, as shown in Fig. 2. Solving the diffusion equation Eq. (2) with boundary conditions Eqs (3–6) one can obtain a concentration field (see Fig. 2a) and a relative growth rate *R* defined as a ratio of the diffusion flows for masked and unmasked surface:

7 $$R=\frac{b}{(l-a)}{\int }_{0}^{l}\frac{\partial C}{\partial z}{|}_{z=b}dx.$$

For an analysis of the relative growth rate for a single NW, one should take the value of *l* ≫ *b* and use the following boundary condition:

8 $$C{|}_{x=l}={C}_{0}.$$

Previous analysis has shown that in the case of selective gas phase epitaxy, the contribution of the increased bulk diffusion to increasing the growth rate is much larger than the surface diffusion and that to find the analytic approximation for *R*_*g*_ only the bulk contribution has to be considered^34,36^.

For a single NW or when the size of the masked surface is much larger than unmasked area, a simplified analytical model can be introduced for the modeling of the growth rate. In this case, lines of equal concentration for the distances smaller than the boundary layer thickness *b* (or smaller than *l*, in the case of periodic array of NWs with period 2 *l*), have circular shape. Thus, in the vicinity of NW, when the bulk concentration *C* depends only on one variable *ρ*, one can describe the diffusion of the precursor in a cylindrical frame as:

9 $$\frac{1}{\rho \,}\frac{\partial }{\partial \rho }(\rho \frac{\partial C}{\partial \rho })=0.$$

Circular shape of isolines in the concentration field holds at the distances *b*/2 corresponding to half of the boundary layer thickness for individual nanowires (or up to half of the period *l* in the case of periodic mask) as shown in Fig. 2a. Since the increase of the growth rate provided by the bulk diffusion is defined by distortion of the concentration field, one can obtain a simplified analytical estimate for *R* in the case of single NW:

10 $$R=\frac{\pi }{2ln(b/w)}\frac{b}{w}$$

while in the case of NWs array (when the thickness of boundary layer *b* should be substituted with *l*, where 2*l* is the periodicity), the relative growth rate *R* reads as follow:

11 $${R}=\frac{{\rm{\pi }}}{2\,\mathrm{ln}(1/{F})}\frac{1}{{F}}.$$

Here we denote the filling factor *F* = *w*/*l*, where *w* is the open area. It is evident that the upper limit for *R* is given by the dependence *R* = 1/*F*. This situation corresponds to the hypothetical case when absorption of the precursor to the mask and the surface diffusion are very effective, i. e. *k* ≫ *l* and *D*^*surf*^ ≫ *D*.

Figure 2b shows the dependencies of *R* obtained by numerical solving of Eq. (2) and by analytical estimate Eq. (11). For comparison, dependence *R* = 1/*F* is also shown. It can be seen that the simplified estimate according to Eq. (11) satisfactorily reproduces the results obtained by the exact numerical solution of the diffusion equation for values of *F* above 0.2; below this value, estimate *R* = 1/*F* is more appropriate to be used. The squares in Fig. 2b show experimental results. Experimental values of the growth rate were obtained by measuring geometrical parameters of NWs at SEM images (see Fig. 1e,f).

It can be seen that experimental values of *R* are smaller than their theoretical estimate, which confirms that the surface diffusion does not provide a noticeable contribution to increase of the growth rate. It can be seen in Fig. 2b that the experimental dependence of *R* saturates at the value of filling factor 1*/F* exceeding 10. This value of *F* corresponds to the size of unmasked surface *w* ~ 1 µm, which is equal to the mean free path of the precursor molecules in the gas. In the case of further increase of the quantity *1/F* (and decrease of *w*), the mass transfer near the unmasked surface is not satisfactorily described by the diffusion model and the relative growth rate *R* saturates.

Thus, the increasing growth rate in the selective area MOVPE process requires changing in TMG flows compared to the traditional planar MOVPE. The theoretical analysis is of great value for growth optimization and improving crystalline quality of the lateral GaN NW as confirmed in the following by characterization.

**Figure 3:**
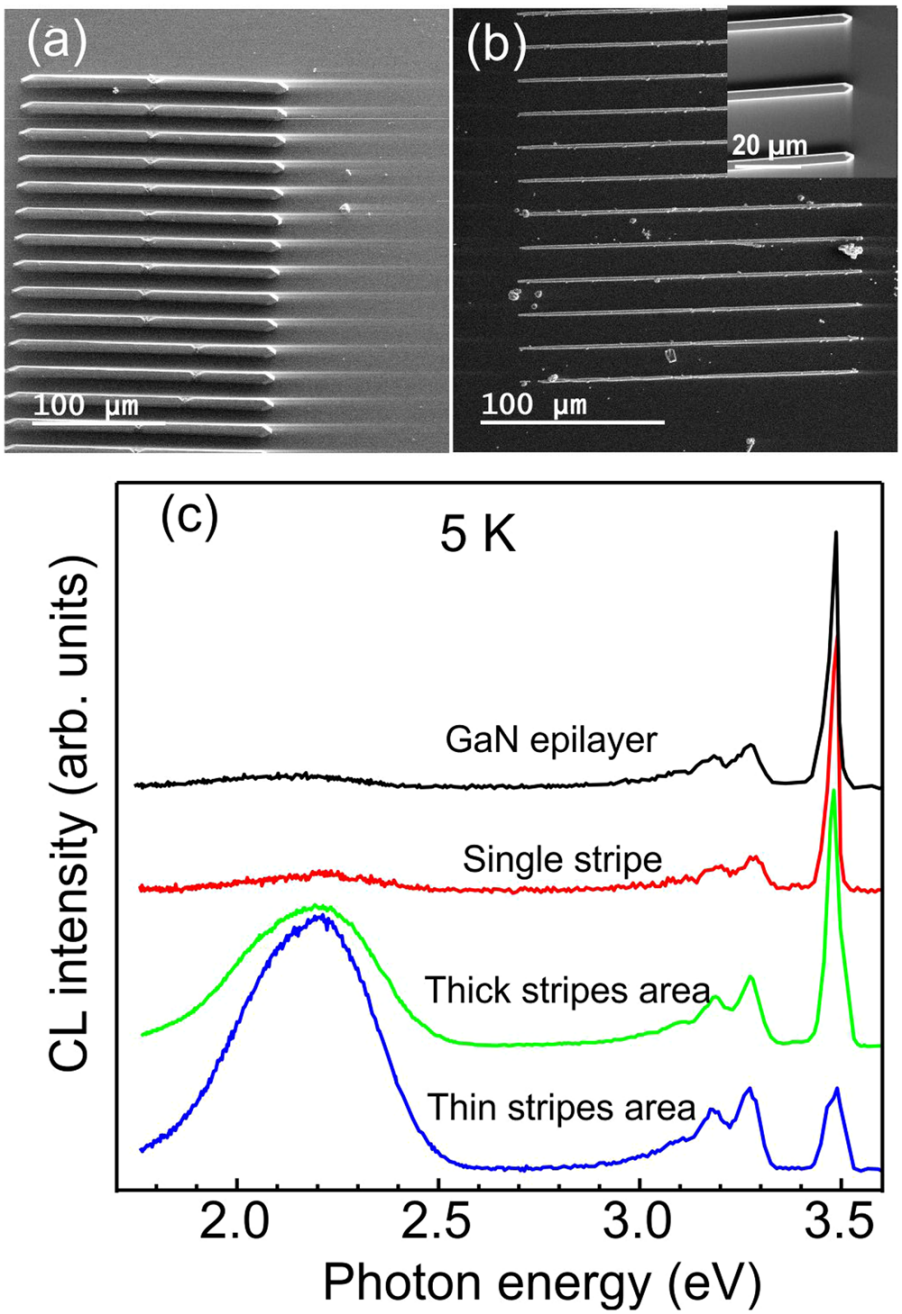
SEM image of the patterned area with GaN planar nanowires with width of 6 µm (**a**) and 2 µm (**b**). The enlarged image of thinner planar stripes is depicted as the inset. (**c**) Low-temperature CL spectra taken from average patterned area (a) and (b) are shown by green and blue lines, respectively. Emission spectrum shown by the red line is measured for a single stripe, i.e. when the electron beam is in the focused mode. CL spectrum measured for the bare epitaxial GaN layer is shown by a black line. Spectra are normalized and shifted vertically for clarity.

We have shown that optimized selective growth allows to achieve an exceptionally high material quality of planar GaN nanowires, which is usually challenging due to difficulties with both keeping a three-dimensional geometrical accuracy during nanostructures formation and requirements for structural perfection, such as a low density of structural and intrinsic point defects; the latter is demanding for optoelectronic and nano-photonic applications. SEM images in Fig. 3a,b show typical patterns with regularly grown rows of planar GaN nanowires having width of 6 and 2 µm, (grown on trenches with initial widths 500 nm and 200 nm) respectively. All planar nanowires have identical shape as can be seen in the inset of Fig. 3b showing enlarged SEM image of the thin stripes.

CL spectra measured at low temperatures are presented in Fig. 3c for the patterned area with thick (green solid line) and thin (blue solid line) nanowires, respectively. In this case, the CL signal has a contribution both from planar GaN nanowires and from the epitaxial layer. For comparison, CL spectra for the bare GaN epitaxial layer and for a single GaN planar nanowire (taken from the stripe top surface) are also shown by black and red lines, respectively. The near band gap emission consists of the peak at ~3.48 eV at 5 K related to the exciton bound to shallow donors (DBE) such as silicon and oxygen and to the donor-acceptor pair emission (DAP) at ~3.28 eV followed by its two LO-phonon replicas^37,38^. Average spectra from the patterned area show also a defect-related band, so-called yellow luminescence (YL), centered at ~2.2 eV. It is important to point out that the CL spectrum taken from the single nanowire is almost identical to the CL spectrum for the epitaxial GaN layer and shows nearly negligible relative intensity of YL, thus, confirming that the fabricated planar GaN nanowires have a high material quality similar to epitaxial layer.

**Figure 4:**
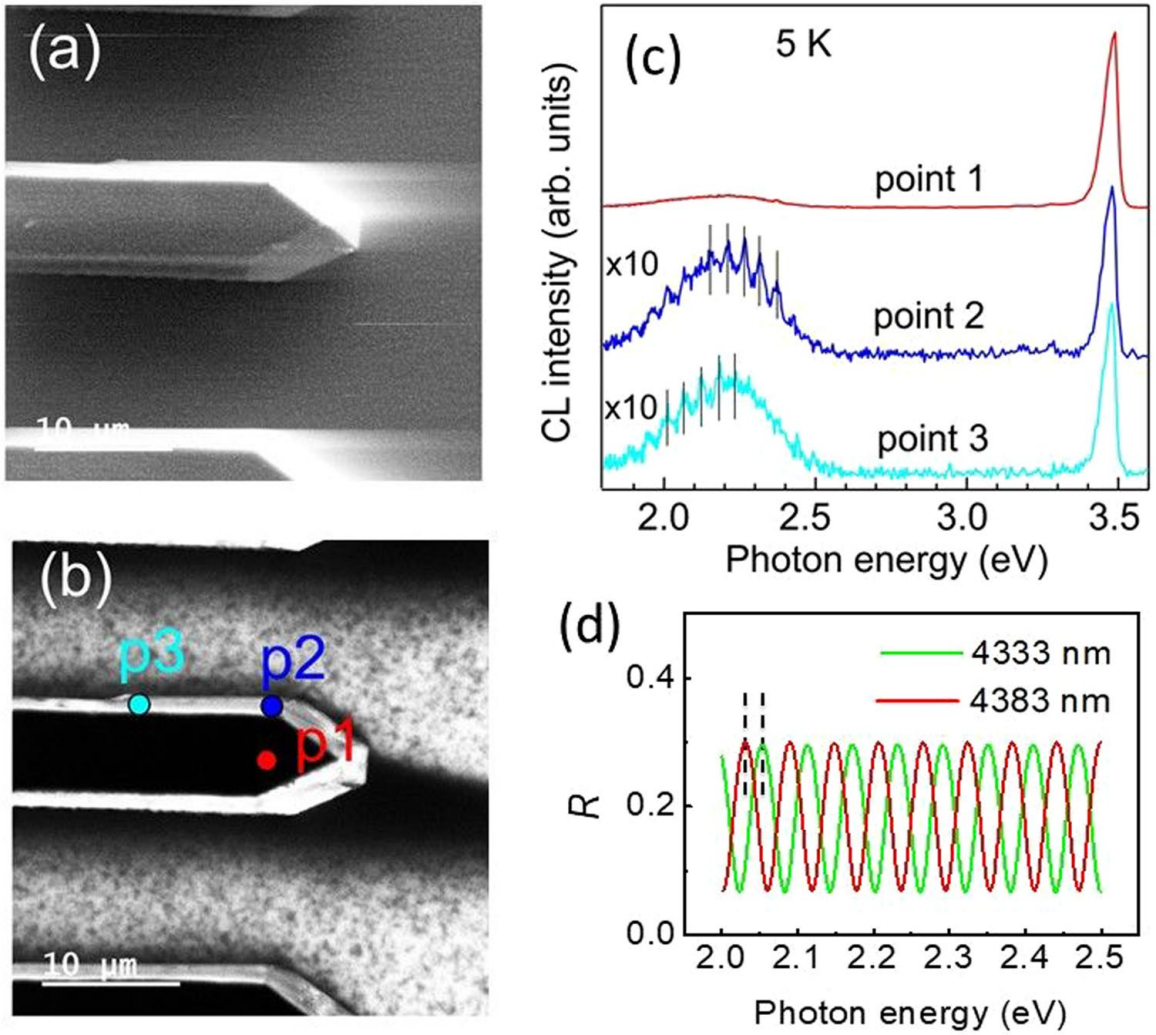
(**a**) SEM and (**b**) panchromatic CL images of the planar GaN nanowires with width of 6 µm. (**c**) Low-temperature CL spectra measured at the same experimental conditions for different points on the stripe as indicated in the panchromatic CL image. Spectra are shifted vertically for clarity. (**d**) reflection spectra calculated according Eq. (12) for the GaN layer grown on sapphire with the layer thickness of 4333 and 4383 nm shown by green and red lines, respectively. Refractive index taken to 2.4 for GaN and 1.7 for sapphire. Vertical dashed lines show the energy shift of ~24 meV between interference maxima obtained for these cases.

Detailed CL measurements at 5 K have been performed for a single planar GaN nanowire illustrated by SEM in Fig. 4a, while a panchromatic CL image taken simultaneously is shown in Fig. 4b. The brighter contrast in CL map corresponds to the higher integrated CL intensity. CL spectra measured in the spot mode at several points as indicated in Fig. 4b are presented in Fig. 4c. As aforementioned, the CL spectrum acquired from the top of the GaN stripe (red line, point 1) is dominated mainly by near band gap excitonic emission. In contrast, CL spectrum measured at the fixed points chosen at the stripe edges have a strong YL emission indicating a higher incorporation of defects during growth on semipolar $\{10\bar{1}2\}$ and/or $\{11\bar{2}2\}$ facets of the planar GaN stripe^39,40^.

It is important also to mention that we have observed a pronounced effect of multiple reflection between top facet of NW (GaN - vacuum) and NW - substrate interface (GaN - sapphire) if the CL spectra are measured at the edges, i.e. at points 2 and 3, respectively, in Fig. 4b. Side view of this NW is shown in Fig. 1f. Several narrow interference maxima at photon energies in the range 1.9–2.5 eV appeared within the broad defect emission band. Note also the step like modification of the tails of the GaN emission at ~3.5 eV, which can be seen at all points 1, 2 and 3.

It is interesting to note that for the point 2 and 3 the peaks are equidistant with energy separation of about 60 meV, but the positions of the peaks for these two points are shifted by ~24 meV. Such behavior indicates that observed peaks are the evidence of the Fabry-Perot mode formation in NW. The interval between different Fabry-Perot mode is determined by ΔE = *πħc*/(*nd*), which gives ~60 meV for the vertical size of NW 4.3 µm (see example in Fig. 1f) calculated with the refractive index n ≈ 2.4 for GaN^41^. The difference between the position of the peaks has the following explanation. The energy of Fabry-Perot modes defines as E = π*ħcM*/(*nd*), where *M* is integer. The observed series of peaks within the yellow band (for the energies between 2.0 eV and 2.5 eV) is characterized by values of M from 33 to 42. Thus, the shift of the peak positions in the yellow band, caused by small variation of the NW thickness *d*, will be more than 30 times larger than the variation of the energy difference between the peaks. Taking into account that the widths of the peaks in the luminescence spectra, acquired in the points 2 and 3 are rather broad, i.e. comparable to the separation between the modes, the difference in the energy separation between the peaks (for spectra measured in points 2 and 3) is not noticeable. This is also illustrated in Fig. 4d, where reflection spectra are calculated for the GaN layers with slightly different thickness of 4333 and 4383 nm, respectively. The reflectance can be calculated using the transfer matrix method^42,43^. For normal incidence we have:

12 $${{R}}_{{\rm{n}}}={|{r}|}^{2}\mathrm{,}$$

where

13 $${r}=\frac{(1-{{n}}_{2})\cos \,\varphi -{i}({{n}}_{2}/{{n}}_{1}-{{n}}_{1})\sin \,\varphi }{(1+{{n}}_{2})\cos \,\varphi -{i}({{n}}_{2}/{{n}}_{1}+{{n}}_{1})\sin \,\varphi }$$

Here the refractive indices for GaN and for sapphire are *n*_*1*_ = 2.4 and *n*_2_ = 1.7, respectively, *ϕ* = ω*n*_1_*d*/*c*. It can be seen that the shift between two peak series of 24 meV is provided by the thickness variation about 1%.

Manifestation of Fabry-Perot modes in the luminescence spectra allows to consider the FIB-MOVPE approach as a method for fabrication of high quality planar cavities with faceted mirrors, which can be used for fabrication of nanophotonic applications.

**Figure 5:**
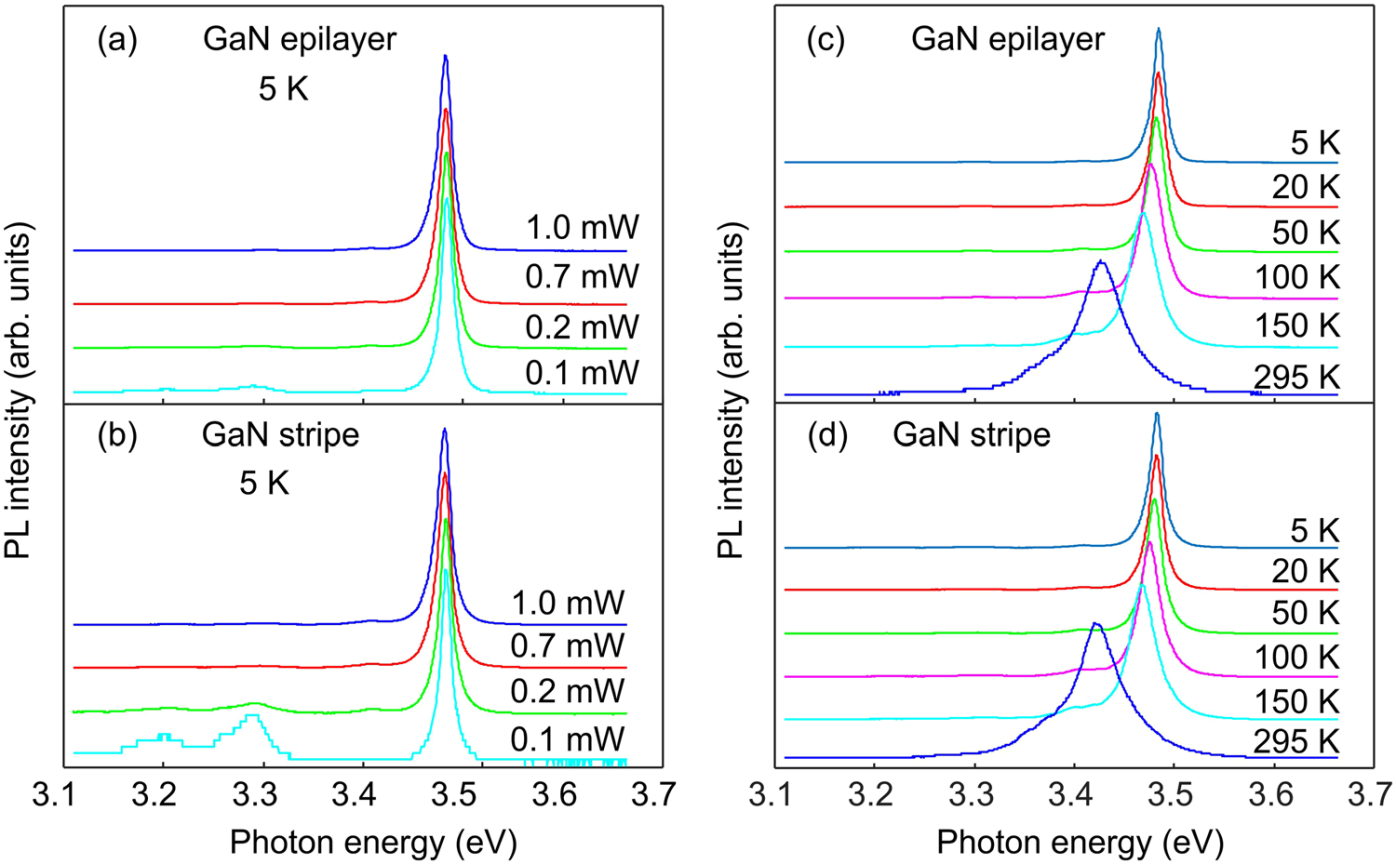
Time-integrated PL spectra taken at 5 K for different excitation power for the bare epitaxial layer (**a**) and for a thin planar GaN nanowire (**b**). Temperature dependent PL spectra for the GaN layer and for the planar GaN nanowire are shown in (**c**) and (**d**), respectively. Spectra are normalized and shifted vertically for convenience.

**Figure 6:**
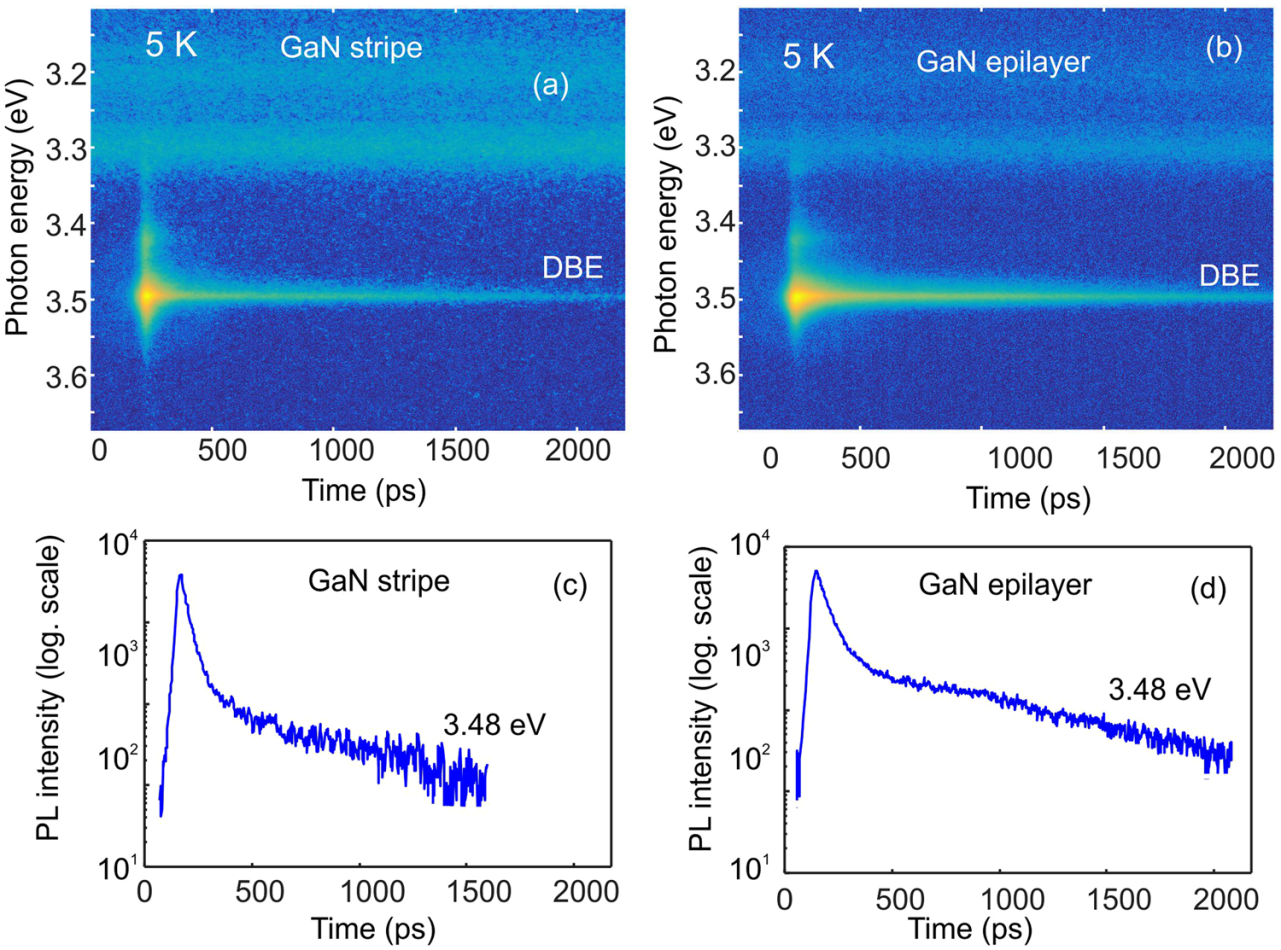
Low temperature TRPL images for (**a**) the GaN epilayer and (**b**) for a single thin planar GaN stripe. (**c**,**d**) correspondent PL decay curves taken at the peak energy of the DBE transition.

The near-band gap PL properties of the planar GaN nanostripes have been studied using a µ-TRPL set-up, where selective excitation by the laser focused to a small spot with a diameter of ~1 µm allows investigation of the emission from a single planar GaN nanowire. Figure 5 shows power and temperature dependent time-integrated PL spectra for a single planar GaN nanowire in comparison with a GaN epitaxial layer. It is obvious that power-dependent and thermal behaviors of the DBE emission measured for GaN stripe and for the bare GaN epilayer are almost identical indicating that the quality of the obtained planar nanowires can be as good as for the 2D layer. The energy position of the DBE line at ~3.48 eV is almost not changing with the excitation power, which is typical for undoped GaN layers^44^; thus, it means that even in planar GaN nanowires the shallow donor concentration is relatively low. The full width at half maximum (FWHM) is slightly changed from ~13 meV to ~16 meV with increasing the excitation power from 0.1 mW to 1 mW. At very low excitation powers, the probability of a donor-accepter pair recombination in unintentionally doped material became higher, which is reflected in an enhanced relative intensity of the emission at ~3.28 eV. At elevated temperatures of 80–100 K the excitonic line is broadened due to the thermalization effect between free exciton and bound exciton states as is clear from Fig. 5c,d and then, the line peak shifts to low energies due to the decrease of the bandgap energy with increasing temperature. Further, the dynamics of charge carriers in semiconductors is directly related to crystal quality and impurity concentrations^45^ making the radiative lifetime of excitons one of the most important characteristics determining a material’s appropriateness for optoelectronic and photonic applications. TRPL images demonstrate rather similar transient behaviors of the DBE transition for the GaN planar nanowires (Fig. 6a) and for the bare GaN epilayer (Fig. 6b).

The PL decay curve in Fig. 6c taken at the peak position of the DBE line measured at a single planar GaN stripe obeys a bi-exponential decay low with the fast and slow recombination rate corresponding to lifetimes of ~50 and ~600 ps, respectively, as extracted by fitting. The DBE kinetics in the case of the GaN layer is also showing a bi-exponential decay behavior with recombination times of ~90 and ~700 ps, respectively. The presence of slow and fast recombination components terms of overlapping between free exciton (XA) transition and DBE. In GaN, the lifetime of free exciton is limited by a non-radiative process even at a low temperature, while the DBE recombination time at low temperatures (5 K) can be considered as radiative lifetime at least in a crystal of very high quality^46^. However, the DBE lifetime in most epitaxial layers is limited by non-radiative recombination mechanisms even at low temperatures^47^. The DBE lifetime is even more severe, suffered by non-radiative recombination in different III-nitride nanostructures due to increased surface-to-volume ratio. From this point of view, studied here planar GaN nanowires show a rather long lifetime of 600 ps for the DBE, which is comparable with the results for the epitaxial GaN layer. This fact can likely be explained by reduced surface-to-volume ratio. Thus, we have shown that the suggested design of the planar GaN nanowires is very promising for potential nanophotonic applications.

In summary, we have optimized the selective area MOVPE process and fabricated high quality lateral GaN NWs by so-called “bottom-up” using FIB patterning of the sapphire substrate. Difficulties in the process are related to increased growth rate in selective area MOVPE compared to conventional lateral MOVPE technique, which without an additional optimization of process parameters results in very low crystalline quality. We have shown that lateral GaN NWs with perfect geometrical shape and of very high crystal quality can be produced by a “bottom-up” approach if the process parameter optimization and Ga precursor concentration is done using theoretical consideration of diffusion of the precursors on the sample surface. High quality of fabricated lateral GaN NWs have been demonstrated through optical properties, which were similar to the properties of the GaN epitaxial layer as confirmed by power- and temperature-dependent near-band gap emission and exciton recombination time. The structure demonstrates pronounced Fabry-Perot modes; thus, the FIB-MOVPE method can be used for fabrication of planar cavities for nanophotonic applications.

## Methods

### Growth

A GaN buffer layer with a thickness of 3 µm was grown by MOVPE on (0001) sapphire wafer. Three-methyl-gallium (TMG) and ammonia (NH_3_) were used as growth precursors. The buffer layer was doped by silicon with a concentration of 2·10^17^ cm^−3^. The doping of the buffer layer is required to prevent electric charging of the wafer by the flow of ions during FIB process, which can defocus the ion beam. An amorphous Si_3_N_4_ mask layer with a thickness of 5 nm was deposited on the top of the buffer layer by MOVPE using silane (SiH_4_) and NH_3_ at the growth temperature of 1000 °C as seen in Fig. 1a.

Ultra-high vacuum FIB technique was employed to etch windows in Si_3_N_4_ mask layer. The scheme of the process is shown in Fig. 1b. The beam of Ga ions with energy 30 keV, has a diameter of 40 nm and a probe current of 450 pA. The exposure dose during etching was 44.8 pC/(µm^2^). For the prevention of re-deposition of the etched materials and formation of Ga droplets, the xenon difluoride (XeF_2_) enhanced etching was utilized, as shown in the Fig. 1b.

The sets of trenches with the length of 200 µm and with the thickness in the interval from 100 to 500 nm were etched. Both, single trenches and periodic arrays (with period 20 μm) of trenches were produced using FIB. After that the patterned wafer was placed into a MOVPE reactor and a selective growth was carried out. Ammonia and TMG were used as the sources of nitrogen and gallium, respectively and hydrogen served as a carrier gas. The flows of TMG and ammonia were 62 micromole/min and 200 cm^3^/min, respectively. The hydrogen pressure was 100 mbar and the substrate temperature was kept at 1030 °C. The flow of hydrogen was 6700 cm^3^/min. The growth process was optimized using theoretical model described above.

### Characterization

Samples were characterized using time-resolved micro-photoluminescence (μ-PL) that was set-up with a spatial resolution of ~1 µm. The third harmonics (λe = 266 nm) from a Ti:sapphire femtosecond pulsed laser with a frequency of 75 MHz has been used as an excitation source. The samples were placed inside a variable temperature (5–300 K) Oxford Microstat allowing X − Y translation with a high precision better than 0.5 µm. Temporal behavior of PL was analyzed using a Hamamatsu synchroscan streak camera with a resolution of ~2 ps.

Samples morphology was studied using a standard Leo 1500 Gemini scanning electron microscope (SEM) combined with a MonoCL4 system allowing CL measurements with a spatial resolution of ~100 nm at an electron beam acceleration voltage of 5 kV. A liquid helium cooled stage could provide temperatures in the range of 5–300 K.

### Data availability

All data generated and/or analyzed during this study are available from the corresponding author on reasonable request.
